# Functional characterization of a novel de novo* CACNA1C* pathogenic variant in a patient with neurodevelopmental disorder

**DOI:** 10.1186/s13041-025-01195-w

**Published:** 2025-03-25

**Authors:** Robin N. Stringer, Xuechen Tang, Bohumila Jurkovicova-Tarabova, Mary Murphy, Klaus R. Liedl, Norbert Weiss

**Affiliations:** 1 Department of Pathophysiology, Third Faculty of Medicine, Charles University, Prague, Czech Republic; 2 Department of General, Inorganic and Theoretical Chemistry, Center for Molecular Bioscience Innsbruck, University of Innsbruck, Innsbruck, Austria; 3 Center of Biosciences, Institute of Molecular Physiology and Genetics, Slovak Academy of Sciences, Bratislava, Slovakia; 4 Department of Biology, Faculty of Education, Trnava University, Trnava, Slovakia; 5 Patient’s Family Representative, Kansas City, Missouri USA

**Keywords:** *CACNA1C*, Ca_v_1.2, Calcium channel, L658P, Channelopathies, Electrophysiology

## Abstract

Mutations in *CACNA1C*, the gene encoding Ca_v_1.2 voltage-gated calcium channels, are associated with a spectrum of disorders, including Timothy syndrome and other neurodevelopmental and cardiac conditions. In this study, we report a child with a de novo heterozygous missense variant (c.1973T > C; L658P) in *CACNA1C*, presenting with refractory epilepsy, global developmental delay, hypotonia, and multiple systemic abnormalities, but without overt cardiac dysfunction. Electrophysiological analysis of the recombinant Ca_v_1.2 L658P variant revealed profound gating alterations, most notably a significant hyperpolarizing shift in the voltage dependence of activation and inactivation. Additionally, molecular modeling suggested that the L658P mutation disrupts interactions within the IIS5 transmembrane segment, reducing the energy barrier for state transitions and facilitating channel opening at more negative voltages. These findings establish L658P as a pathogenic *CACNA1C* variant primarily associated with severe neurological dysfunction and expands the phenotypic spectrum of *CACNA1C*-related disorders.

**Supplementary Information:**

The online version contains supplementary material available at 10.1186/s13041-025-01195-w.

## Main text

Ca_v_1.2 calcium channels are highly expressed in the cardiac, neuronal, and endocrine systems, where they play important roles in regulating excitation–contraction coupling, neurotransmitter release, hormone secretion, and gene transcription [1, 2]. First mutations in the *CACNA1C* gene, which encodes Ca_v_1.2, have been implicated in a multisystem disorder known as Timothy syndrome (TS). TS is characterized by prolonged cardiac QT intervals, life-threatening arrythmias, neurological and developmental abnormalities, and features of autism spectrum disorders [3, 4]. Since the initial identification of TS-causing mutations, the spectrum of *CACNA1C*-related disease variants has expanded significantly, encompassing a broad range of clinical phenotypes. While some mutations lead to complex, multisystem disorders resembling TS, others result in more localized pathologies, with patients presenting predominantly with either cardiac or neurological symptoms [5].

**Fig. 1:**
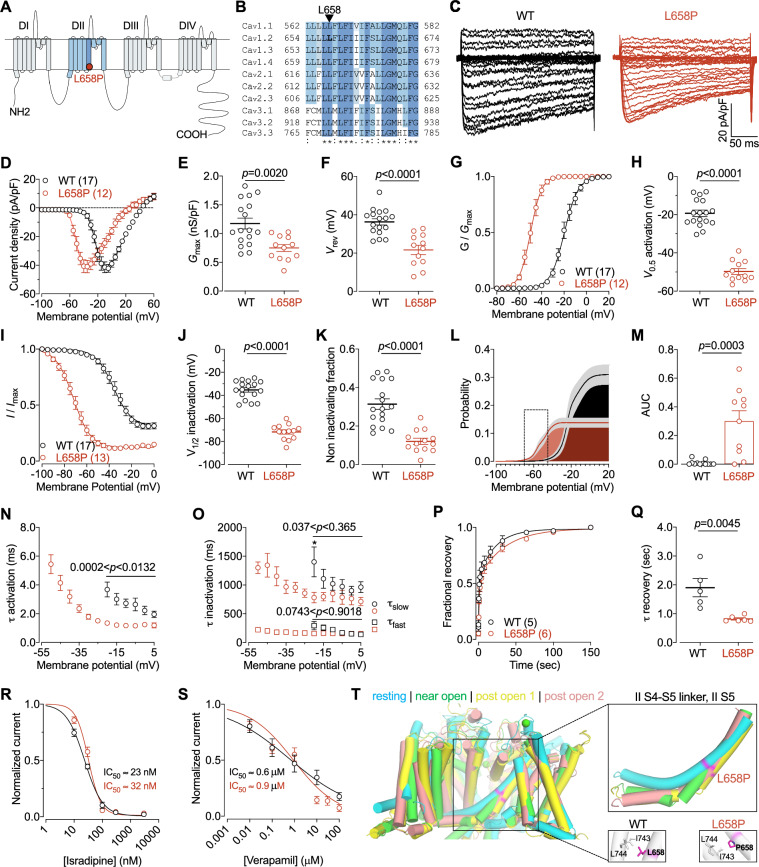
Electrophysiological properties of the Ca_v_1.2 L658P variant. **A** Location of the L658P missense variant (red dot) within the secondary membrane topology of Ca_v_1.2 channel. **B** Amino acid sequence alignment of the IIS5 transmembrane segment showing the conservation of the L658 residue across the 10 human voltage-gated calcium channel isoforms. Alignments were performed using UniProt (Ca_v_1.1 Q13698; Ca_v_1.2 Q13936; Ca_v_1.3 Q01668; Ca_v_1.4 O60840; Ca_v_2.1 O00555; Ca_v_2.2 Q00975; Ca_v_2.3 Q15878; Ca_v_3.1 O43497; Ca_v_3.2 O95180; Ca_v_3.3 Q9P0X4). **C** Representative L-type current traces recorded from tsA-201 cells expressing Ca_v_1.2 wild-type (WT, black traces) and L658P variant channels (red traces), in combination with Ca_v_β_2_, and Ca_v_α_2_δ_1_. **D** Corresponding mean current–voltage (*I*/*V*) relationship. **E** Mean maximal macroscopic conductance (*G*_max_) obtained from the fit of the *I*/*V* curves with the modified Boltzmann Eq. (1). **F** Mean reversal potential (*V*_rev_) values. **G** Mean normalized voltage dependence of activation. **H** Mean half-activation potential (*V*_0.5_ activation) values obtained from the fit of the activation curves with the modified Boltzmann Eq. (2). **I** Mean normalized voltage dependence of steady state inactivation. **J** Mean half-inactivation potential (*V*_0.5_ inactivation) values obtained from the fit of the inactivation curves with the modified Boltzmann Eq. (3). **K** Mean non-inactivating channel fraction values. **L** Mean window currents calculated from the activation and inactivation curves using the Eq. (4). The squared area shows the window current between − 70 and − 55 mV, corresponding to a range of neuronal resting membrane potentials. **M** Mean window current values calculated from the area under the curve between − 70 and − 55 mV. **N** Mean time constant τ values of current activation kinetics. **O** Mean time constant τ values of current inactivation kinetics. **P** Mean normalized recovery of inactivation kinetics. **Q** Mean time constant τ values of recovery from inactivation obtained by fitting the recovery curves with a single-exponential Eq. (5). **R** Dose–response of Ca_v_1.2 channels for isradipine. **S** Dose–response of Ca_v_1.2 channels for verapamil. **T** Molecular modeling of Ca_v_1.2 showing the impact of the L658P mutation on various superimposed Ca_v_1.2 channel states including resting (cyan), near-open (green), and high-voltage inactivated states (yellow and orange)

In this study, we report a 2.5-year-old boy presenting with a complex phenotype, including refractory epilepsy, global developmental delay, hypotonia, hypoxemia, oral-pharyngeal dysphagia, restrictive ventilatory defect, hydronephrosis, visual impairment, epileptic encephalopathy, mitochondrial respiratory chain defects, micrognathia, and single transverse palmar crease. Brain MRI revealed diffusely prominent subarachnoid spaces and supratentorial ventricles, consistent with diffuse brain underdevelopment and/or parenchymal volume loss, as well as a simplified gyral pattern indicative of impaired neuronal proliferation. Additionally, small cavities in the left temporal lobe and right cerebellar hemisphere were observed, consistent with encephalomalacia likely resulting from past insults. Despite these systemic abnormalities, electrocardiograms were generally normal. Whole exome sequencing identified a de novo heterozygous missense variant (c.1973T > C) in *CACNA1C*, resulting in the substitution of leucine at position 658 with proline (L658P). This mutation is located within the highly conserved transmembrane IIS5 segment of the Ca_v_1.2 voltage-gated calcium channel (Fig. 1A and B) [6]. This variant has not been reported in the Genome Aggregation Database (gnomAD), but was reported in a cross-section study of the neuropsychiatric phenotypes associated with *CACNA1C*-related disorders [7]. Furthermore, in silico prediction tools have classified this variant as pathogenic [8].

To investigate the functional impact of the L658P variant, the mutation was introduced into the human Ca_v_1.2 channel using site-directed mutagenesis. Recombinant wild-type (WT) and L658P channels were heterologoulsy expressed in tsA-201 cells, and patch-clamp recordings were performed to assess their biophysical properties (see Supplementary methods). Both WT and L658P-expressing cells exhibited typical voltage-activated L-type currents (Fig. 1C and D). The maximal macroscopic conductance was significantly reduced by 36% (*p* = 0.0020) in cells expressing the L658P variant (0.75 ± 0.06 nS/pF, n = 12) compared to cells expressing the WT channel (1.17 ± 0.09 nS/pF, n = 17) (Fig. 1E). Additionally, a hyperpolarizing shift in the reversal potential by −14.7 mV was observed, from 36.3 ± 1.6 mV in WT cells to 21.7 ± 2.4 mV in L658P-expressing cells (*p* < 0.0001) (Fig. 1F). Strikingly, the L658P mutation caused a pronounced hyperpolarizing shift in the voltage dependence of activation of Ca_v_1.2 (Fig. 1G). Specifically, the mean half-activation potential was shifted by − 30.4 mV (*p* < 0.001), from − 19.3 ± 1.7 mV (n = 16) in WT cells to − 49.7 ± 1.5 mV (n = 12) in L658P-expresing cells (Fig. 1H). Similarly, the voltage dependence of steady state inactivation was shifted by − 36.5 mV, from −35.3 ± 1.9 mV (n = 17) in WT cells to − 71.7 ± 1.9 mV (n = 13) in cells expressing the L658P variant (*p* < 0.001) (Fig. 1I and J). This shift was accompanied by a significant reduction in the non-inactivating channel fraction, from 0.31 ± 0.03 (n = 17) in WT cells to 0.12 ± 0.02 (n = 13) in L658P-expressing cells (*p* < 0.0001) (Fig. 1K). Importantly, the pronounced hyperpolarizing shift in activation and inactivation resulted in a hyperpolarizing shift in the window current (Fig. 1L). This created a significant window current at hyperpolarized membrane potentials in cells expressing the L658P variant, which was absent in WT cells (Fig. 1M). The L658P mutation also slightly accelerated the activation kinetics of the L-type current (Fig. 1N), while inactivation kinetics remained largely unaltered (Fig. 1O). Notably, our recordings were performed using barium as the charge carrier, preventing the assessment of calcium-dependent inactivation of Ca_v_1.2 [9]. Consequently, it is possible that the L658P mutation exhibits a different effect on channel inactivation in the presence of calcium. Furthermore, the L658P variant caused a 2.3-fold reduction in the time constant of recovery from inactivation, from 1.90 ± 0.32 s (n = 5) in WT cells to 0.82 ± 0.04 s (n = 6) (*p* = 0.0045) (Fig. 1P and Q). Lastly, we assessed whether the L658P mutation affected the pharmacological sensitivity of the Ca_v_1.2 channel to therapeutic drugs. No significant differences in the IC50 values for isradipine (Fig. 1R) or verapamil (Fig. 1S) were observed between WT and L658P channels. Altogether, these findings demonstrate that the L658P mutation induces profound gating alterations in the Ca_v_1.2 channel, most notably characterized by a significant hyperpolarizing shift in the voltage dependence of activation and inactivation. This shift reflects both gain- and loss-of-function effects, respectively, ultimately leading to the emergence of a window current at hyperpolarized membrane potentials.

To begin elucidating the molecular mechanisms by which the L658P mutation induces gating defects, we conducted molecular modeling of the L658P variant in various Ca_v_1.2 channel states (resting, near-open, and post-open) (see Supplementary methods). The combined structural models were aligned based on the voltage-sensing domains in domain II (VSD II) and domain III (VSD III), with the exclusion of the S4 segments to minimize alignment-related influences on the structural divergence points. Structurally, we hypothesized that the L658P mutation facilitates transitions between channel states, contributing to the hyperpolarizing shift in the voltage dependence of activation and inactivation. As shown by the combined view of different Ca_v_1.2 channel states, the L658P variant is located at the structural divergence point of the modeled states (Fig. 1T). Specifically, this mutation appears to enhance the mobility of the lower S5 segment and the connecting S4-S5 linker in domain II, effectively lowering the energy barriers for state transitions and enabling channel activation a lower voltage. In the WT channel, the leucine residue at position 658 is tightly anchored by hydrophobic interactions with neighboring residues on IIS6. Replacing this leucine with proline disrupts these interactions, loosening the helix due to the loss of a backbone hydrogen bond. This structural flexibility is likely to reduce the energy required for transitions, thereby enhancing voltage sensitivity. In the WT channel, such transitions would typically require initiation by VSD II, a process that is rendered more energetically favorable by the presence of the proline residue in the L658P mutated channel.

In conclusion, this study identifies the L658P mutation as a pathogenic *CACNA1C* variant with profound effects on Ca_v_1.2 channel gating. The unique biophysical alterations observed emphasise the complex interplay between gain- and loss-of-function mechanism in *CACNA1C*-related disorders. Notably, despite the patient’s severe neurological impairment, electrocardiograms remained normal, distinguishing the L658P mutation from variants typically associated with long-QT syndrome [7]. The absence of a cardiac phenotype may be attributed to differential expression and regulation of Ca_v_1.2 in neuronal versus cardiac tissues [10]. It is also possible that cardiac-specific splicing within the region containing the mutation could explain the lack of a cardiac phenotype. However, the observation that the L658P mutation causes a combined hyperpolarizing shift in both the voltage dependence of activation and inactivation—making the channel more likely to be open at resting membrane potentials—suggests an overall gain-of-function effect. Further investigations using tissue-specific expression models in both neuronal and cardiac settings will be essential to fully elucidate the impact of the L658P mutation and its exact role in the pathophysiology of *CACNA1C*-related disorders.

## Supplementary Information

Supplementary Material 1.

## Data Availability

All data generated or analyzed during this study are included in this published article.

## Abbreviations

MRI: Magnetic resonance imaging
TS: Timothy syndrome
VSD: Voltage sensing domain
WT: Wild-type
