# Supplementary material for: Functional characterization of a novel de novo CACNA1C pathogenic variant in a patient with neurodevelopmental disorder
Source: Mol Brain. 2025 Mar 25;18:26. doi: 10.1186/s13041-025-01195-w (PMC11934713; doi:10.1186/s13041-025-01195-w)
Supplement: Supplementary file 1 — Supplementary Material 1. [file 13041_2025_1195_MOESM1_ESM.docx]

**Functional characterization of a novel *de novo* *CACNA1C* pathogenic variant in a patient with neurodevelopmental disorder**

Robin N. Stringer^1^, Xuechen Tang^2^, Bohumila Jurkovicova-Tarabova^3,4^, Mary Murphy^5^, Klaus R. Liedl^2^, Norbert Weiss^1,3^

**Supplementary information**

**Supplementary** **materials and methods**

***Plasmid cDNA constructs and site-directed mutagenesis***

The L658P Ca_v_1.2 variant was generated by site directed mutagenesis performed by GenScript using the wild-type (WT) human Ca_v_1.2 as template. The fidelity of the construct was confirmed by full-length sequencing of the coding region.

***Cell culture and heterologous expression***

Human embryonic kidney tsA-201 cells were grown in DMEM medium supplemented with 10% fetal bovine serum and 1% penicillin/streptomycin (all media purchased from Invitrogen). And maintained under standard condition at 37 ^o^C in a humidified atmosphere containing 5% CO_2_. Heterologous expression was performed by transfecting cells with 5 μg of plasmid cDNAs encoding for the human Ca_v_1.2, Ca_v_β_2a_, and Ca_v_α_2_δ_1_ in a ratio 3:1:1, and empty pEGFP plasmid as transfection marker using the calcium/phosphate method.

### *Patch clamp electrophysiology*

Patch clamp recordings of L-type currents in tsA-201 cells expressing the WT and L658P Ca_v_1.2 variant were performed 72 h after transfection in the whole-cell configuration at room temperature (22–24 °C) in a bath solution containing (in millimolar): 10 BaCl_2_, 125 CsCl, 1 MgCl_2_, 10 D-glucose, 10 HEPES, adjusted to pH 7.4 with CsOH. Patch pipettes were filled with a solution containing (in millimolar): 110 CsCl, 3 Mg-ATP, 0.5 Na-GTP, 2.5 MgCl_2_, 5 D-glucose, 10 EGTA, and 10 HEPES, adjusted to pH 7.4 with CsOH, and had a resistance of 2–4MΩ. The calculated liquid junction potential was − 2.6 mV and therefore was not corrected from the recordings. Recordings were performed using an Axopatch 200B amplifier (Axon Instruments) and acquisition and analysis were performed using pClamp 10 and Clampfit 10 softwares, respectively (Axon Instruments). The linear leak component of the current was corrected using a P/4 subtraction protocol and current traces were digitized at 10 kHz and filtered at 2 kHz.

The voltage dependence of Ca_v_1.2 channel activation was assessed by measuring the peak L-type current in response to 250 ms depolarizing steps, ranging from -100 mV to +60 mV in 5 mV increments, from a holding membrane potential of -100 mV. The current-voltage (*I*/*V*) relationship was fitted with the following modified Boltzmann equation 1:

$$\left( 1 \right) I\left( V \right)= Gmax \frac{(V-Vrev)}{1+ \exp\frac{(V0.5-V)}{ka}}$$

with *I*(*V*) being the peak current amplitude at the command potential *V*, *G*_max_ the maximum macroscopic conductance, *V*_rev_ the reversal potential, *V*_0.5_ the half-activation potential, and *k* the slope factor. The voltage dependence of the whole-cell Ca_v_1.2 channel conductance was calculated using the following modified Boltzmann equation 2:

$$\left( 2 \right) G\left( V \right)= \frac{Gmax}{1+ \exp\frac{(V0.5-V)}{ka}}$$

With *G*(*V*) being the macroscopic conductance at the command potential *V*. The voltage dependence of steady-state inactivation was assessed by measuring the peak L-type current amplitude in response to a 25 ms depolarizing step to 0 mV, following a 5-s conditioning prepulse ranging from -100 mV to 20 mV in 5 mV increments. The current amplitude during each test pulse was normalized to the maximal current amplitude and plotted as a function of the prepulse potential. The voltage dependence of the steady-state inactivation was fitted with the following two-state Boltzmann equation (3):

$$\left( 3 \right) I\left( V \right)= \frac{(1-C)}{1+ \exp\frac{\left( V-V0.5 \right)}{ki}}+C$$

with *I*_max_ corresponding to the maximal peak current amplitude, *V*_0.5_ to the half-inactivation voltage, and *C* to a constant. The window current was calculated from combining the conductance and steady-state inactivation of each cell and was fitted with the following function (4):

$$\left( 4 \right) I\left( V \right) G\left( V \right)= \frac{1}{1+ \exp\frac{\left( -\left( V0.5-V \right) \right)}{ka}} \frac{(1-C)}{1+ \exp\frac{\left( V-V0.5 \right)}{ki}}+C$$

Recovery from inactivation was evaluated by applying a 5-s depolarizing prepulse at 0 mV (inactivating prepulse) to maximize the proportion of inactivated channels, followed by a 25 ms test pulse at -10 mV after varying intervals ranging from 1 ms to 15 s at -100 mV (interpulse). The peak current during the test pulse was expressed as a ratio of the maximum prepulse current and plotted against the interpulse interval. The data were fitted with the following single-exponential function (5):

$$\left( 5 \right) \frac{I}{Imax}= A (1-{exp}^{\left( -\frac{t}{} \right)})$$

where τ is the time constant for channel recovery from inactivation.

***Molecular modeling***

Structural models of various Ca_v_1.2 channel states were constructed using cryo-EM structures of Ca_v_1.2 captured in their respective states. Alignments were carefully verified and corrected using highly conserved residues in the Ca_v_1.2 subunit (uniport accession: Q13936-4) to ensure high-quality homology modeling, performed with MOE (Molecular Operating Environment, v2022.02, Chemical Computing Group ULC, Montreal, QC, Canada). To optimize the initial homology models, energy minimization was performed using the Amber10:EHT force field with periodic boundary conditions (AMBER 10, University of California, San Francisco, CA, USA, 2008). The modeled structures were color-coded based on the voltage of the experimental state in which the template structure was captured, relative to the channel’s *V*_max_. The color gradient ranged from cyan to orange, representing the transition from the resting state (cyan) to the inactivated state at high voltage (orange). The Na_v_1.7 structure stabilized by the protoxin-II (PDB accession code: 6N4I) [9] served as the template for the resting state model (cyan). The near-open state model (green) was based on the original cryo-EM structure of Ca_v_1.2 (PDB accession code: 8WE6) [10]. High-voltage inactivated states (yellow and orange) were modeled using the Ca_v_1.1 structure (PDB accession code: 5GJW) [11] and the Ca_v_3.1 structure (PDB accession code: 6KZO) [12] to represent even higher voltage conditions. To analyze structural variations across states, the modeled structures were superimposed with relatively voltage-sensing domains VSD I and VSD II near the L658P mutation site, excluding their respective S4 segments. This approach minimized alignment-related artifacts, ensuring accurate assessment of structural changes associated with different states.

***Statistics***

Data values are presented as mean ± S.E.M for *n* measurements. Student’s t test (unless stated otherwise) was carried out to test for statistical significance and data sets were considered different for *p* < 0.05.
